# Efficacy of assessing circulating cell-free DNA using a simple fluorescence assay in patients with triple-negative breast cancer receiving neoadjuvant chemotherapy: a prospective observational study

**DOI:** 10.18632/oncotarget.23520

**Published:** 2017-12-21

**Authors:** Kwonoh Park, Miyoung Woo, Jeong Eun Kim, Jin-Hee Ahn, Kyung Hae Jung, Jin Roh, Gyungyub Gong, Sung-Bae Kim

**Affiliations:** ^1^ Department of Oncology, Asan Medical Center, University of Ulsan College of Medicine, Seoul, Republic of Korea; ^2^ Medical Oncology and Hematology, Department of Internal Medicine, Pusan National University Yangsan Hospital, Yangsan-si, Republic of Korea; ^3^ Department of Pathology, Asan Medical Center, University of Ulsan College of Medicine, Seoul, Republic of Korea

**Keywords:** cell-free DNA, fluorescence assay, triple-negative breast cancer, neoadjuvant chemotherapy

## Abstract

This study aims to assess cell-free DNA (CFD) by a fluorescence assay as a biomarker for early prediction of a pathologic complete response (pCR) and relapse in patients with triple-negative breast cancer (TNBC) undergoing neoadjuvant chemotherapy. Patients with clinical stage II or III TNBC scheduled for neoadjuvant chemotherapy were prospectively enrolled. All patients underwent four cycles of Adriamycin plus cyclophosphamide (AC), followed by four cycles of cisplatin or docetaxel chemotherapy and surgery. Blood samples were obtained before the initial chemotherapy (baseline-CFD) and after four AC neoadjuvant chemotherapy cycles (AC-CFD) to evaluate CFD levels. In total, 72 patients who met the inclusion criteria were enrolled. The mean baseline-CFD and AC-CFD levels were 239 ± 68 and 210 ± 66 ng/mL, respectively, with a significant decline in the CFD levels after AC neoadjuvant chemotherapy (*P* = 0.001). In the 33.6-month median follow-up, 18 cases of relapse were reported. A ROC curve analysis of baseline-CFD was performed to determine the predictive value for relapse, and an area under the curve of 0.62 (95% CI, 0.46–0.78) at 264 ng/mL was obtained. Patients with baseline-CFD >264 ng/mL were at a higher risk of relapse than those with baseline-CFD ≤264 ng/mL (HR, 2.84; 95% CI, 1.11–7.24; *P* = 0.029). Multivariate analysis established baseline-CFD as an independent predicting factor for relapse (HR, 3.74; 95% CI, 1.32–10.53; *P* = 0.013). In conclusion, baseline-CFD measured by a fluorescence assay might be a potential biomarker to predict relapse, which could be useful for risk stratification of TNBC.

## INTRODUCTION

Triple-negative breast cancer (TNBC) is defined by the lack of an estrogen receptor (ER), a progesterone receptor (PR), and a human epidermal growth factor receptor-2 (HER-2). TNBC has an unfavorable prognosis because of its aggressive tumor biology and its lack of response to endocrine treatment or HER2 blockade. Reportedly, neoadjuvant chemotherapy is a mainstay treatment for locally advanced TNBC [1], leading to overall survival rates equivalent to those of adjuvant chemotherapy. The advantages of neoadjuvant chemotherapy over adjuvant chemotherapy comprise the possibility of less extensive surgery, such as a breast-conserving operation (BCO), and *in vivo* prediction ability about the response to chemotherapy [2]. Conversely, the disadvantages of neoadjuvant chemotherapy are as follows: in the case of poor response, patients are at a risk of delayed optimal time for surgery, and in the case of favorable response, especially achievement of the pathologic complete response (pCR) in the early phase, patients are at a risk of overtreatment. Hence, it is important and active area of research for early prediction during neoadjuvant chemotherapy rather than late confirmation after neoadjuvant chemotherapy, to facilitate chemotherapy adjustment based on individual patient’s response to optimize efficacy and reduce treatment toxicity.

The pCR is regarded as a surrogate endpoint of long-term clinical outcome that estimate the efficacy of neoadjuvant chemotherapy [3], which is even more prominent in patients with TNBC than non-TNBC [1, 3]. Although pCR is considered as a surrogate marker of efficacy of neoadjuvant chemotherapy [4], it has a limitation for early prediction of neoadjuvant chemotherapy due to taking several months for confirming pCR. Therefore, non-invasive and robust biomarkers that can be used for early prediction of response to neoadjuvant chemotherapy are currently indicated.

Considering the aggressive tumor biology, poor prognosis, and paradoxically favorable chemosensitivity associated with TNBC [3, 5], additional postoperative chemotherapy, including patients treated with standard preoperative treatment, could be selected in clinical practice or research [6, 7]. To date, these prognostic markers for additional postoperative chemotherapy have been primarily based on the results of surgical specimens such as residual disease after the completion of standard neoadjuvant chemotherapy [6]. However, a high proportion of relapses systemically occurring in TNBC, such as in the viscera [1, 8], could be attributed to micrometastasis. Therefore, additional prognostic biomarkers could facilitate risk stratification of relapse and reflect systemic tumor burden, which might be essential for adopting additional postoperative chemotherapy.

The detection of circulating cell-free DNA (CFD) in the plasma or serum reveals some characteristics of a potential biomarker candidate for tumor response and detection. Arguably, CFD is associated with apoptosis, necrosis, and active release of cancer cells in the tumor microenvironment and is reportedly released from necrotic or apoptotic non-tumor cells phagocytosed by macrophages or other scavenger cells [9, 10]. Since its discovery in 1977 [11], CFD is considered as a “liquid biopsy” that could be used for several applications such as detection, follow-up, and response to various malignancies; moreover, it is convenient for obtaining repeated blood samples without invasive biopsies [12–18]. However, CFD assays used to date are both labor intensive and expensive because of complex processes such as DNA extraction from blood and DNA concentration measurement by quantitative PCR [19]. Thus, CFD assays have been confined to research laboratories with limited application in the clinical practice. Recently, CFD assays that use a convenient and simple fluorescence-based method to evaluate biological samples directly without a complicated DNA extraction process have been developed [14]. This novel technique demonstrates a correlation between CFD levels and both disease progression and death in patients with colorectal and breast cancer [13, 20].

This study aims to establish the role of CFD using the novel method in patients with TNBC who underwent neoadjuvant chemotherapy. We evaluated the association of CFD levels with early prediction of achieving pCR and investigated whether CFD could be used as a prognostic biomarker for predicting relapse in patients with TNBC.

## RESULTS

### Baseline characteristics

Between April 2012 and December 2014, among 88 patients with TNBC enrolled in the PACER (NCT02001519) study, we assessed CFD levels of 61 patients before and after undergoing Adriamycin (doxorubicin) plus cyclophosphamide (AC) neoadjuvant chemotherapy. In addition, CFD levels were evaluated for additional 11 among 34 patients with TNBC enrolled in the Neo-Shorter (NCT02001506) study. Overall, we enrolled 72 patients in this study who fulfilled the inclusion criteria (Supplementary Figure 1). Table 1 provides the details of patients and their tumors. Based on the final pathologic report on surgical specimens, five patients (ER-positive, four; HER-2-positive, one) transitioned from TNBC to non-TNBC.

**Table 1:** Baseline characteristics of patients

Characteristics	*N*	Percent
**Age (years, range)**	46 (25–71)	
**ECOG**		
0	68	94%
1	4	6%
**Ki-67 (median, range)**	70 (0–90)	
**Histologic grade**		
1	0	0
2	26	36%
3	46	64%
**Tumor stage**		
1	5	7%
2	43	60%
3	23	32%
4	1	1%
Tumor size (mm, median, range)^*^	39.5 (13–89)	
**Node stage**		
0	19	27%
1	31	43%
2	8	11%
3	14	19%
**Clinical stage**		
IIA	14	19%
IIB	27	38%
IIIA	17	24%
IIIB	14	19%
**CA 15-3 (U/mL, median, range)**^†^	9.8 (2.3–42.8)	
≤30	65	94%
>30	4	6%
**TILs (%)**^‡^		
≤40	53	77%
>40	16	23%

ECOG, Eastern Cooperative Oncology Group; TILs, tumor-infiltrating lymphocytes.

^*^Tumor size was primarily calculated using magnetic resonance imaging.

^†^CA 15-3 was based on 69 patients with available data.

^‡^TILs were assessed using the initial pathologic slide, which was available in 69 patients.

### Treatment and tumor response

All 72 patients completed four AC cycles. Among these patients, 16 underwent surgical treatment after the initial four AC cycles [complete response (CR), 10 patients; progressive disease (PD), two; and consent withdrawal, four] and 56 were treated with AC followed by cisplatin or docetaxel chemotherapy. Of the 56 patients, 46 completed four cisplatin or docetaxel chemotherapy cycles as planned, nine were terminated because of PD, and one discontinued therapy after two cisplatin chemotherapy cycles because of prolonged cytopenia without any evidence of disease progression. All 72 patients underwent surgical treatment with curative intent. Among these, 48 were treated with BCO and 24 with modified radical mastectomy (MRM). The median interval time between the completion of neoadjuvant chemotherapy and surgery was 29 days (range, 19–55 days). Responses to AC were as follows: CR (10 patients, 14%), PR (49 patients, 69%), stable disease (SD; 11 patients, 16%), and PD (2 patients, 3%). Pathologic responses were as follows: pCR (17 patients, 24%) and non-pCR (55 patients, 76%; Supplementary Table 1).

### Baseline-CFD and baseline characteristics

The baseline-CFD level of healthy controls at 170 ± 10 ng/mL was significantly lower than that of all patients (239 ± 68 ng/mL; *P* = 0.001) and that of 20 patients with similar age of <40 years (220 ± 54 ng/mL; *P* = 0.001; Supplementary Table 2).

We conducted a correlation analysis between baseline-CFD and baseline characteristics, including age, histologic grade, tumor size (T stage), LN involvement (N stage), and clinical stage. Baseline-CFD was not associated with initial tumor characteristics such as T stage, N stage, or histologic grade (T stage 1-2 vs. 3, *P* = 0.313; N stage 0 vs. 1–3; *P* = 0.317; and histologic grade 2 vs. 3, *P* = 0.997). Additionally, no correlation was observed between baseline-CFD and CA15-3 or Ki-67 levels (CA15-3, <20 U/mL vs. ≥20 U/mL; *P* = 0.227 and Ki-67, 0%–20% vs. 30%–100%; P-value=0.580), where as statistically significant differences were observed between baseline-CFD and tumor-infiltrating lymphocyte (TIL) levels (TILs, ≤40% vs. >40%; *P* = 0.027; Table 2).

**Table 2:** A correlation analysis of baseline-CFD^*^ and baseline characteristics

Characteristics	*N*	Baseline-CFD^*^ (ng/mL, mean)
**Age (years)**		
≤40	21	221.9
>40	51	246.2
		0.125
**Tumor stage**		
1–2	48	233.3
3–4	24	250.7
*P*		0.301
**Node stage**		
0	18	253.2
1–3	54	234.4
*P*		0.445
**Clinical stage**		
IIA-IIB	41	241.9
IIIA-IIIB	31	235.5
*P*		0.692
**Histologic grade**		
2	26	239.1
3	46	239.1
*P*		0.997
**Ki-67 (%)**		
0–20	9	251.1
30–100	63	237.4
*P*		0.580
**TILs (%)**^†^		
≤40	53	250.2
>40	16	204.7
*P*		0.027

CFD, cell-free DNA; TILs, tumor-infiltrating lymphocytes.

^*^Baseline-CFD indicates CFD levels evaluated before neoadjuvant chemotherapy.

^†^TILs were assessed using the initial pathologic slide, which was available for 69 patients.

### CFD and response to neoadjuvant chemotherapy

**Figure 1:**
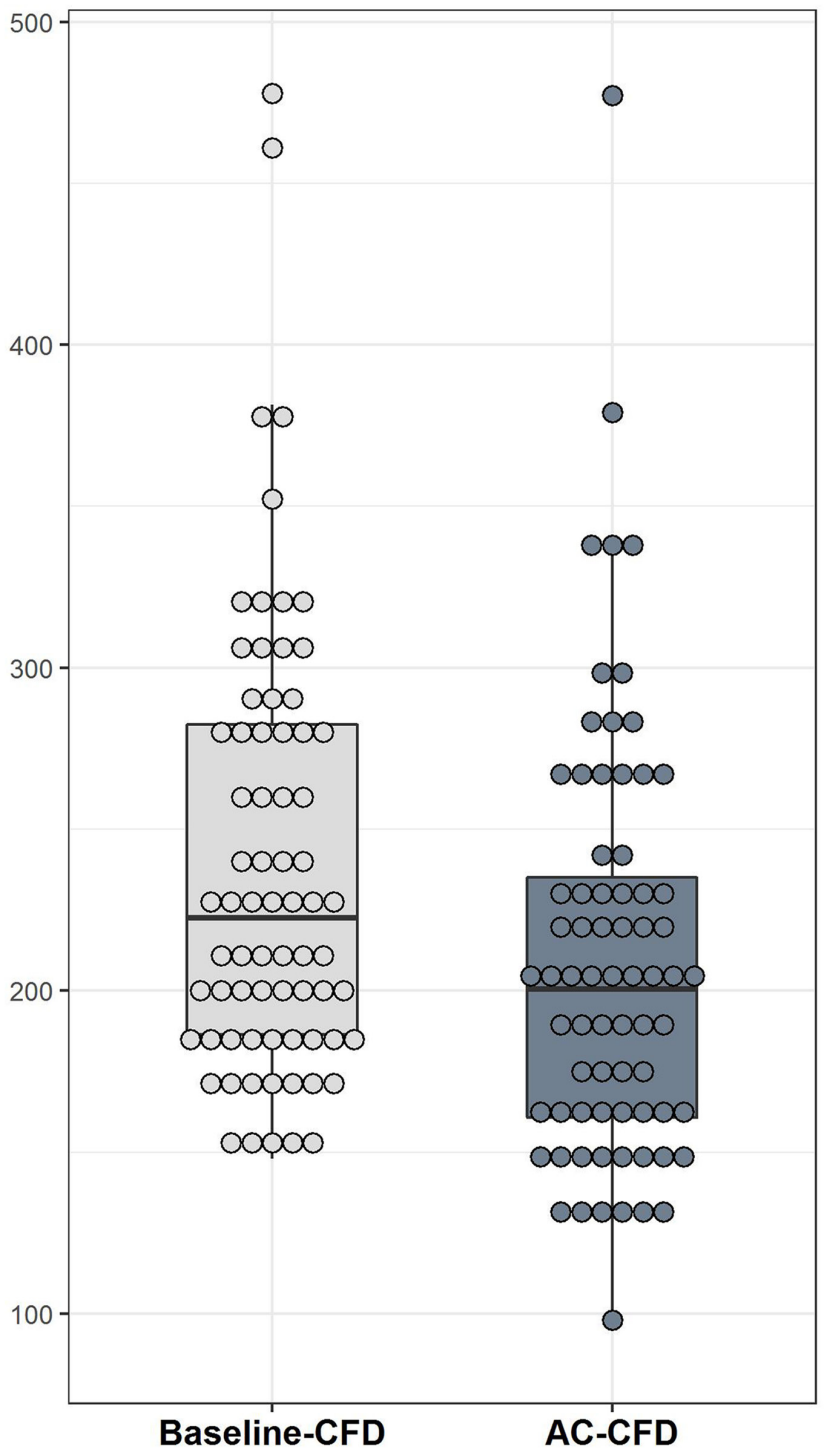
The CFD level significantly decreased after AC chemotherapy (*P* = 0.001) Baseline-CFD and AC-CFD indicate the CFD levels measured before and after four cycles of AC, respectively. Box plots show medians and 25^th^ and 75^th^ percentiles; dot plots show the individual marker distribution.

In this study, the mean baseline-CFD and AC-CFD levels were 239 ± 68 and 210 ± 66 ng/mL, respectively, with a considerable decline in CFD levels after AC chemotherapy (*P* = 0.001; Figure 1). Considering the radiologic response to AC chemotherapy, no statistically significant differences were observed between patients who responded and those who did not in relation to baseline-CFD levels, AC-CFD levels, and changes in CFD levels (*P* = 0.814, 0.881, and 0.927, respectively). Regarding the pathologic response, no statistically significant differences were observed between responders and non-responders in relation to baseline-CFD levels, AC-CFD levels, and changes in CFD levels (*P* = 0.937, 0.500, and 0.570, respectively; Table 3). In a subgroup with aggressive tumor biology (histologic grade 3, 46 patients), with marginal significance, AC-CFD levels substantially declined in pCR patients than in non-pCR patients, with higher variations seen in CFD levels of pCR patients [AC-CFD levels, pCR (183) vs. non-pCR (219); *P* = 0.090 and changes in CFD levels, pCR (−62.3) vs. non-pCR (−17.9); *P* = 0.133; Table 4]. Regarding the evaluation of other markers, pCR patients witnessed an upsurge in Ki-67 levels compared with non-pCR patients (*P* = 0.022). However, no differences were observed between both patient groups regarding TILs and baseline CA15-3 levels (*P* = 0.980 and 0.463, respectively; Table 3).

**Table 3:** Association among each CFD levels, Ki-67, baseline CA15-3, radiologic/pathologic response to neoadjuvant chemotherapy, and relapse

Characteristics	*N*	Baseline-CFD (mean)	AC-CFD (mean)	Change in CFD (mean)	Ki-67 (mean)	CA 15-3 (mean)	TILs^†^
**Response to AC**							
PR or CR	59 (82%)	240.0	211.2	−28.8	61.9	12.0	30.2
NC or PD	13 (18%)	235.0	208.2	−26.9	66.2	13.8	19.6
*P*		0.813	0.881	0.927	0.599	0.549	0.120
**Pathologic response**							
pCR	17 (24%)	237.9	201.1	−36.8	73.5	11.3	28.3
Non-pCR	55 (76%)	239.5	213.6	−25.9	59.3	12.7	28.2
*P*		0.937	0.500	0.570	0.022	0.463	0.980
**Relapse**							
Non-relapse	54 (75%)	232.7	203.9	−28.7	61.8	12.7	31.0
Relapse	18 (25%)	258.5	230.8	−27.6	65.0	11.3	20.0
*P*		0.161	0.137	0.920	0.632	0.500	0.192

AC, Adriamycin plus cyclophosphamide regimen; CFD, cell-free DNA; CR, complete response; SD, stable disease; pCR, pathologic complete response; PD, progressive disease; PR, partial response; TILs, tumor-infiltrating lymphocytes.

^*^Baseline-CFD and AC-CFD indicate levels of CFD measured before and after four cycles of Adriamycin plus cisplatin chemotherapy, respectively. Change in CFD indicates the difference between AC-CFD and baseline-CFD.

^†^TILs were assessed using the initial pathologic slide, which was available for 69 patients.

**Table 4:** Associations between each CFD level and achievement of pCR according to histologic grade (grade 2 and grade 3)

	Histologic grade 2			Histologic grade 3		
	pCR	Non-pCR	*P*	pCR	Non-pCR	*P*
**Number**	5	21		12	34	
Baseline-CFD^*^	219.8	243.7	0.753	245.5	236.9	0.762
AC-CFD^*^	244.2	204.9	0.205	183.2	219.0	0.090
Change in CFD^*^	+24.4	−38.8	0.064	−62.3	−17.9	0.133

pCR, pathologic complete response.

^*^Baseline-CFD and AC-CFD indicate levels of CFD were assessed before and after four cycles of Adriamycin plus cisplatin chemotherapy, respectively. Change in CFD indicates the difference between AC-CFD and baseline-CFD.

### CFD and prognosis

**Figure 2:**
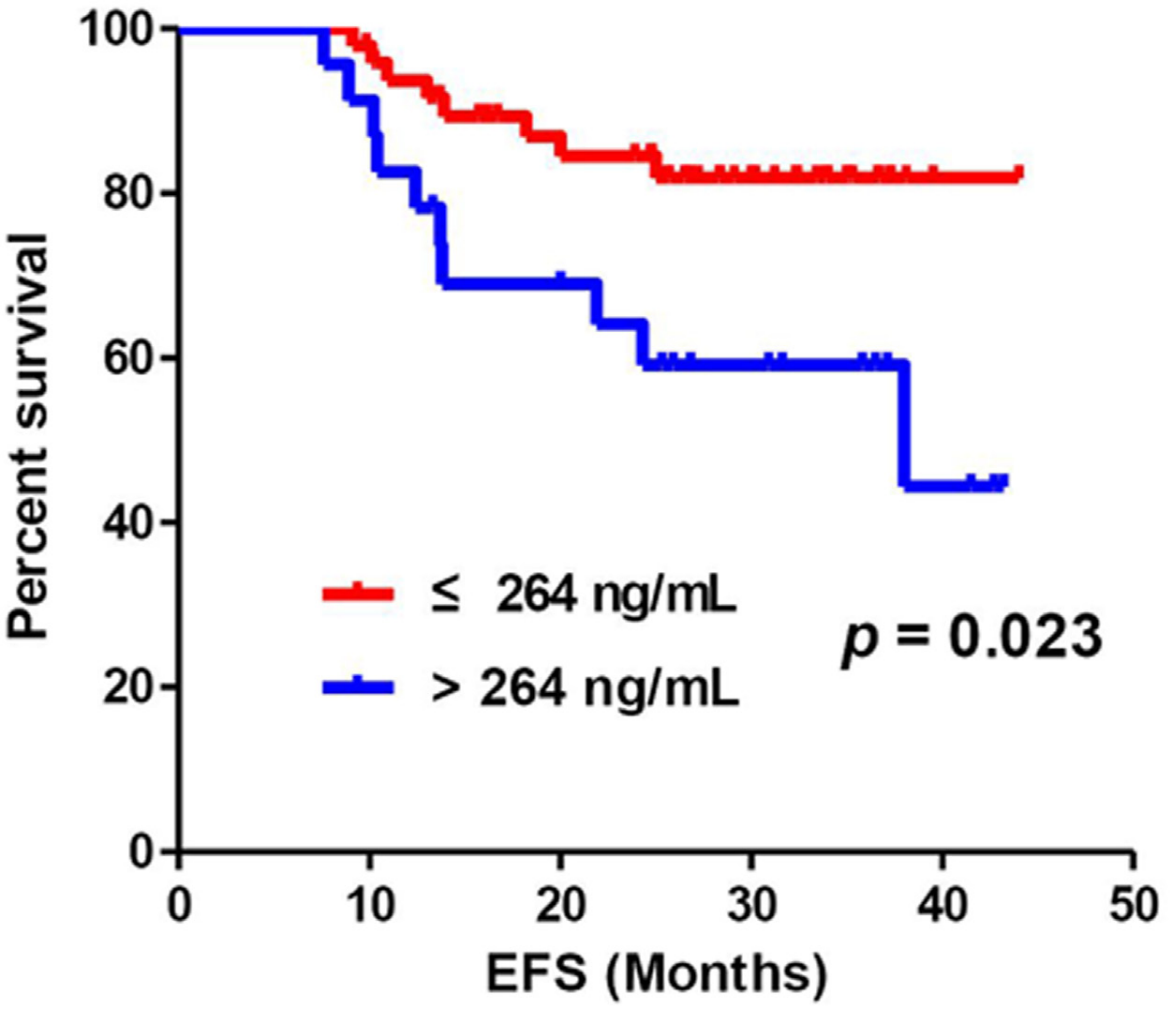
There was a significant difference of EFS according to the cutoff level of baseline-CFD, 264 ng/mL (*P* = 0.023)

In this study, we reported 18 relapse cases in the 33.6-month median follow-up period. We observed an increasing trend toward higher baseline-CFD levels in patients who relapsed, although it was not statistically significant (relapse, 259 ng/mL; non-relapse, 233 ng/mL; *P* = 0.161; Table 3). We conducted a receiver operating characteristic (ROC) curve analysis of baseline-CFD levels for relapse and observed an area under the curve of 0.62 (95% CI, 0.46–0.78; Supplementary Table 3). With a cutoff of 264 ng/mL, the diagnostic power of CFD had a specificity of 76% and a sensitivity of 56%. The patients Patients with baseline-CFD levels >264 ng/mL demonstrated higher relapses than those with baseline-CFD levels ≤264 ng/mL (HR, 2.84; 95% CI, 1.11–7.24; *P* = 0.029; Figure 2). In the univariate analysis, baseline-CFD and response to AC were relevant factors for predicting relapse, whereas other clinical and pathologic parameters such as tumor size, node stage, clinical stage, histologic grade, and TILs were not relevant. In the multivariate analysis, baseline-CFD and response to AC were independent clinical parameters related to event-free survival (EFS; Table 5).

**Table 5:** Univariate and multivariate analyses of event-free survival

Variable	Univariate		Multivariate^*^	
	Hazard ratio	*P*	Hazard ratio	*P*
**Baseline-CFD (≤264 vs. >264)**	2.84 (1.11–7.24)	0.029	3.74 (1.32–10.53)	0.013
**Age (≤40 vs. > 40)**	1.15 (0.43–3.12)	0.782	0.381 (0.13–1.13)	0.081
**Tumor stage (T1-2 vs.T3)**	0.94 (0.35–2.51)	0.901		
**Node stage (N0-1 vs. N2-3)**	1.88 (0.54–6.58)	0.318		
**Clinical stage (II vs. III)**	1.89 (0.75–4.79)	0.181		
**Histologic grade (2 vs. 3)**	0.91 (0.35–2.36)	0.846		
**Response to AC** (CR or PR vs. SD or PD)	5.27 (2.05–13.54)	0.001	5.53 (2.06–14.88)	0.001
**Pathologic response** (pCR vs. non-pCR)	2.30 (0.53–10.04)	0.270		
**Ki-67 (≤20% vs. ≥ 30%)**	1.32 (0.30–5.91)	0.717		
**TILs (≥ 40% vs. <40%)**	5.32 (0.71–40.1)	0.105	3.43 (0.44–26.95)	0.241

AC, Adriamycin plus cyclophosphamide regimen; CFD, cell-free DNA; CR, complete response; SD, stable disease; pCR, pathologic complete response; PD, progressive disease; PR, partial response; TILs, tumor-infiltrating lymphocytes

^*^In the multivariate analysis, while the clinical stage was included, tumor stage and node stage were excluded.

## DISCUSSION

The present study evaluated the potential role of CFD for predicting the outcomes of treatments such as neoadjuvant chemotherapy in TNBC patients using an innovative and simple technique, i.e., a direct fluorescence method. The results of this study cohort preliminarily confirmed the potential ability of baseline-CFD for predicting a relapse. To the best of our knowledge, this is the fourth study to investigate the use of the fluorescence-based CFD assay for cancer [13, 14, 20] and the first to investigate the efficacy of this assay in a preoperative setting for breast cancer.

The study also showed that CFD levels significantly decreased after chemotherapy. In line with the results of previously reported PCR assays [12, 19, 21] and fluorescence method [13], this study suggests that a fluorescence-based CFD assay could reflect tumor burden. Unfortunately, we could not establish a correlation between baseline-CFD and other baseline gross tumor burden-related characteristics, such as tumor size or LN involvement, in this study. These discordant results could be partly attributed to the small sample size of our study. However, even previous studies have not identified a significant relationship between baseline-CFD and tumor characteristics [22, 23]. The results of this study suggest that because other factors, such as tumor biology or non-gross tumor lesion (e.g., micrometastasis), could be involved in the CFD levels, further research is warranted to elucidate these conflicting findings.

The predictive value of CFD, measured by PCR assay, has been demonstrated in several studies investigating lung cancer [21], rectal cancer during neoadjuvant chemoradiotherapy [24], and breast cancer [15, 16]; some of these studies have also reported a correlation between declining CFD levels and early treatment response. However, despite an overall decline in CFD levels, as mentioned earlier, we did not observe a significant relationship between the changes in CFD levels and the radiologic or pathologic responses to neoadjuvant chemotherapy. Nevertheless, in patients with high-grade tumors, the reduction in CFD levels was higher in pCR patients than in non-pCR patients. These results are in corroboration with previous studies, highlighting that patients with TNBC subtype were more sensitive to neoadjuvant chemotherapy and attained a higher rate of pCR than non-TNBC patients [3]. Moreover, this assertion is further supported by a previous report that illustrates higher rates of pCR achievement based on the increasing histologic grade of TNBC [25]. Despite the significant decrease of CFD after chemotherapy, the lack of correlation between CFD and response to AC or inadequate association between CFD and pCR achievement were unexpected findings. This result can be explained as follows: (a) our study might have a methodological limitation because of a relatively small sample size, (b) decline in non-gross tumor lesions, such as micrometastasis, unrelated to the radiologic response might have adversely affected the CFD levels, (c) chemotherapy might have induced transient increase in the CFD levels [26], and (d) the CFD levels were measured after completing AC chemotherapy and not after overall completion of neoadjuvant chemotherapy; thus, it might not have reflected accurate pCR achievement.

Of note, baseline-CFD was an independent prognostic factor for the prediction of relapse. Although the present study did not identify the tumor stage, node stage, clinical stage, or histologic grade as prognostic factors, it demonstrated that the performance of fluorescence-based CFD assays could indicate an independent prognostic biomarker. Assumedly, CFD levels not only represent the gross tumor burden but also systemic tumor burden, including micrometastasis or tumor biology, based on our results that CFD was an independent prognostic factor for predicting a relapse and that CFD levels significantly declined after chemotherapy, even though CFD was unrelated to the baseline characteristics and the radiologic response to AC. Recently, postoperative additional chemotherapy such as comprising 6–8 cycles of capecitabine, have demonstrated improved results in patients with residual disease after the completion of standard neoadjuvant chemotherapy [6]. Based on our results, we hypothesized that baseline-CFD might be helpful in identifying patients at a high risk of relapse and in planning additional adjuvant chemotherapy.

This study had several limitations that restricted its possible practicality as a definite biomarker for TNBC. First, the measurement processes of fluorescence-based CFD assays was not adequately validated. Although PCR-based CFD assays have been used since a long time, the validation of CFD measurement methods, such as reproducibility for comparability and clinical utility, has not be established to date [27]. However, a major technical issue related to prior PCR-based CFD assays was the reliability of the DNA extraction phase, which allows only small amounts of DNA to be obtained from plasma or serum, and CFD levels appear to vary widely across different studies. Because the fluorescence-based CFD assay used in this study evaluates CFD levels directly without a DNA extraction phase, it can avoid the concern regarding the reliability of the DNA extraction phase. The results of the study by Goldshtein et al. supported this method by demonstrating that a fluorescence method could minimize intra- and day-to-day variations in assays [14]. Second, because the sample size of this study comprised a relatively small number of patients, only statistical trends were observed in most outcomes. However, we established that CFD levels were statistically significant as independent prognostic factors. The lack of statistical significance of other crucial prognostic factors, such as clinical stage or TILs, might be explained by the same limitation. The number of healthy controls was also inadequate in this study. Third, we did not conduct consecutive assessments of CFD levels upon the completion of neoadjuvant chemotherapy and after surgical treatment. Perhaps, an extensive investigation of sequential CFD level measurements throughout the overall perioperative treatment duration could elucidate the following: the CFD biology, whether CFD levels increased transiently during chemotherapy, and whether CFD levels could more precisely predict prognosis based on the relationship between the postoperative residual CFD levels and EFS. Hence, it is obvious that this study was primarily exploratory. Nevertheless, this study of a homogeneous cohort succeeded in elucidating a possible role of CFD as a predictive and prognostic biomarker using a simple and inexpensive fluorescence-based CFD assay, thereby generating a hypothesis for more extensive future studies.

In conclusion, this prospective explorative study indicated that baseline-CFD levels obtained using a simple and convenient fluorescence assay could predict relapses, suggesting the usefulness of baseline-CFD as a prognostic biomarker for risk stratification of relapse in patients with TNBC. In addition, changes in CFD levels have the potential to predict pCR achievement, particularly in patients with TNBC with aggressive tumor biology. Larger prospective studies are warranted to clarify the advantages of including this method as a reliable marker for determining additional postoperative treatment.

## MATERIALS AND METHODS

### Study design and patients

This study was conducted as a substudy of two studies (NCT02001519, PACER and NCT02001506, Neo-Shorter) that were conducted to evaluate the efficacy of neoadjuvant chemotherapy for locally advanced breast cancer. For these two studies, we enrolled patients with clinical stage II or III (tumor size, >1.5 cm or LN size, >1.5 cm) breast cancer scheduled for neoadjuvant chemotherapy, including AC. Between April 2012 and December 2014, patients with TNBC subtype from whom blood samples were obtained to assess CFD levels before and after four AC cycles were enrolled in this study. TNBC was defined by the absence of ER, PR, and HER-2. ER and PR were assessed by immunohistochemical (IHC) analyses (ER: clone 6F11, NOVO, Newcastle, UK; PR: clone 16, NOVO) and HER-2 was assessed by either fluorescence *in situ* hybridization (FISH), silver *in situ* hybridization (SISH), or IHC (clone 4B5; VENTANA, Tucson, AZ). ER/PR negativity was defined as the presence of <1% of tumor cells with a positive nuclear staining or Allred score of 0–2, and HER-2 negativity was defined as IHC 0, IHC 1+, and negative FISH or SISH in the case of IHC 2+. The subtype was determined on the basis of the initial pathologic results obtained before the onset of neoadjuvant chemotherapy. Patients who changed from the TNBC subtype to the non-TNBC subtype based on the analysis of a surgical specimen after neoadjuvant chemotherapy were also included in the present study. Other requirements for enrollment of patients were as follows: the Eastern Cooperative Oncology Group performance status 0-1; no history of prior anticancer treatment (e.g., radiotherapy, chemotherapy, hormonal therapy, and biologic agents); adequate bone marrow, renal, liver, and heart functions; and intact cognitive function for understanding and providing written informed consent. Patients who showed no evidence of a primary tumor (T0) or had a previous history of heart problems, such as anthracyclines being contraindicated, were excluded from the study. Clinical staging was performed according to the American Joint Committee on Cancer guidelines [28]. For other pathologic assessments, Ki-67 IHC was locally estimated by IHC using the Mib-1 monoclonal antibody (Dako, Glostrup, Denmark). Evaluations of the quantity and location of TILs were performed using full-face hematoxylin and eosin-stained sections, exactly as previously described, and were independently assessed by two pathologists (K Gong and J Rho) who were blinded to the clinical outcomes; the mean value of the two assessments was used for further analyses [29]. Both Ki-67 and TILs levels were reported as continuous variables (per 10% increments).

Furthermore, we compared CFD levels in patients with TNBC with healthy controls and obtained blood samples from five healthy female individuals without a history of cancer and acute or chronic illness at the time of medical checkup to evaluate the performance of CFD using the fluorescence method. The study protocol was reviewed and approved by the institutional review board of Asan Medical Center, Seoul, South Korea; each patient provided written informed consent.

### Treatment and follow-up

The enrolled patients underwent laboratory testing, mammography, breast ultrasound, and breast MR scan before initiating neoadjuvant chemotherapy, which comprised four AC cycles (60 mg/m^2^ of Adriamycin plus 600 mg/m^2^ of cyclophosphamide) followed by four cisplatin (75 mg/m^2^; NCT02001519) or docetaxel (75 mg/m^2^; NCT02001506) chemotherapy cycles. After the completion of each cycle, we conducted a physical examination using caliper. Breast ultrasound and mammography were performed at both the completion of AC chemotherapy and before surgery, and when the physical examination suspected any progression. We performed breast MR scans before surgical treatment. When CR or PD was identified by clinical or radiologic examination after the four AC neoadjuvant chemotherapy cycles, the patients underwent surgical treatments such as BCO or MRM with sentinel or axillary LN dissection. In other cases, patients with PR or SD underwent surgical treatments following the four planned cisplatin or docetaxel chemotherapy cycles. Patients who changed from TNBC to non-TNBC after neoadjuvant chemotherapy and surgical treatment were postoperatively treated with interventions such as hormone therapy or trastuzumab treatment depending on their final pathologic results. After the completion of treatment, patients underwent a follow-up of clinical and radiologic examinations every 3 months during the first year, every 6 months during the second year, and annually in the subsequent years.

### CFD assay

Plasma samples were obtained from patients within 7 days starting the initial neoadjuvant chemotherapy (baseline-CFD) and after 14 days following the completion of four AC neoadjuvant chemotherapy (AC-CFD) cycles. The difference between AC-CFD and baseline-CFD levels was used for further analysis (designated as “change in CFD”). A volume of five millimeters of peripheral blood was drawn into an ethylenediaminetetraacetic acid-coated tube before physical examination or biopsy. Blood samples were incubated at 4°C and transferred to the study laboratory within 4 h for processing. Plasma samples were obtained by centrifuging the peripheral blood at 3000 *×g* for 10 min. The plasma samples were collected from the upper layer of the supernatant and stored in aliquots at −80°C.

The CFD assay directly detected CFD from patients’ blood samples. The SYBR Gold Nucleic Acid Gel Stain (Molecular Probe) was diluted first at 1:1000 in dimethyl sulfoxide (Sigma-Aldrich, UK) and then at 1:8 in phosphate-buffered saline (Gibco). DNA solutions (10 μL) were applied to black 96-well plates (CoStar). Diluted SYBR Gold (40 μL) was added to each well (final dilution, 1:10,000). Fluorescence was measured using a 96-well fluorimeter (Victor X3; PerkinElmer) at an emission wavelength of 535 nm and an excitation wavelength of 485 nm.

### Statistical analysis

The clinical endpoints of this study investigated the correlation between each CFD level (baseline-CFD, AC-CFD, and change in CFD) and pCR achievement and between each CFD level and EFS. EFS was defined as the time from study enrollment to the first occurrence of disease progression, which resulted in inoperability, distant metastasis, or death from any cause. Patients who survived without any event until the cutoff date (January 2016) were censored at the last follow-up. The overall radiologic response rate was defined as the proportion of patients with CR or partial response among patients with evaluable lesions for response according to the Response Evaluation Criteria in Solid Tumors ver. 1.1 [30]. pCR was determined by microscopic examinations of excised tumors and LNs after completing chemotherapy and was defined as no residual invasive cancer in either [31].

Descriptive statistics used to summarize the characteristics of the study population are reported as proportions and medians. Student’s *t*-test or Mann–Whitney *U* test was performed to analyze each CFD level between the two groups. The area under the ROC curve was calculated to evaluate the predictive performance of continuous variables and to determine the cutoff value for discrimination of recurrence. The Kaplan–Meier product-limit method was used to analyze EFS. To identify the independent role of CFD in predictive prognosis, univariate and multivariate analyses using Cox proportional hazards regression analysis were performed, with a backward selection procedure used for model selection. All statistical analyses were two sided and were performed using the SPSS version 19.0 (SPSS Inc. Chicago, IL). *P* < 0.05 was considered as statistically significant.

## SUPPLEMENTARY MATERIALS FIGURE AND TABLES



## Abbreviations

BCO: breast-conserving operation
CFD: cell-free DNA
CR: complete response
EFS: event-free survival
ER: estrogen receptor
HER-2: human epidermal growth factor receptor-2
pCR: pathologic complete response
PD: progressive disease
PR: progesterone receptor
SD: stable disease
TILs: tumor-infiltrating lymphocytes
TNBC: triple-negative breast cancer
