# Supplementary material for: Efficacy of assessing circulating cell-free DNA using a simple fluorescence assay in patients with triple-negative breast cancer receiving neoadjuvant chemotherapy: a prospective observational study
Source: Oncotarget. 2017 Dec 21;9(3):3875–86. doi: 10.18632/oncotarget.23520 (PMC5790507; doi:10.18632/oncotarget.23520)
Supplement: Supplementary file 1 [file oncotarget-09-3875-s001.pdf]

Efficacy of assessing circulating cell-free DNA using a simple fluorescence assay in patients with triple-negative breast cancer receiving neoadjuvant chemotherapy: a prospective observational study

SUPPLEMENTARY MATERIALS

Supplementary Table 1: Neoadjuvant chemotherapy and outcomes

Characteristics	Number	Percent
**Neoadjuvant chemotherapy process**		
AC	16	22%
AC plus Cisplatin	45	63%
AC plus Docetaxel	11	15%
**Surgery**		
BCO	48	67%
MRM	24	33%
**Response to AC**		
CR	10	14%
PR	49	69%
SD	11	16%
PD	2	3%
**Response to Cisplatin or Docetaxel***		
CR	6	11%
PR	39	70%
SD	2	4%
PD	9	16%
**Pathologic response**		
pCR	17	24%
Non-pCR	55	76%

AC, Adriamycin plus cyclophosphamide regimen; BCO, breast-conserving operation; CR, complete response; MRM, modified radical mastectomy; SD, stable disease; pCR, pathologic complete response; PD, progressive disease; and PR, partial response.
*Fifty-six patients were treated with subsequent cisplatin or docetaxel.

Supplementary Table 2: Comparisons of baseline-CFD levels in healthy controls and patients

Characteristics	Total patients	Patients aged <40 years	Healthy controls
**Number of participants**	72	20	5
**Age**	46	34.4	34.8
**Baseline-CFD (ng/mL)**	239 ± 68	220 ± 54	170 ± 10

Supplementary Table 3: Receiver operating characteristics curve analysis for relapse

Characteristics	Area under curve (95% CI)
**Baseline-CFD**	0.62 (0.46–0.78)
**AC-CFD**	0.60 (0.46–0.75)
**Change in CFD**	0.51 (0.34–0.78)
**Tumor stage**	0.52 (0.37–0.67)
**Node stage**	0.58 (0.43–0.73)
**Clinical stage**	0.61 (0.47–0.75)
**Histologic grade**	0.48 (0.33–0.64)
**Ki-67**	0.52 (0.37–0.67)
**TILs**	0.40 (0.26–0.54)

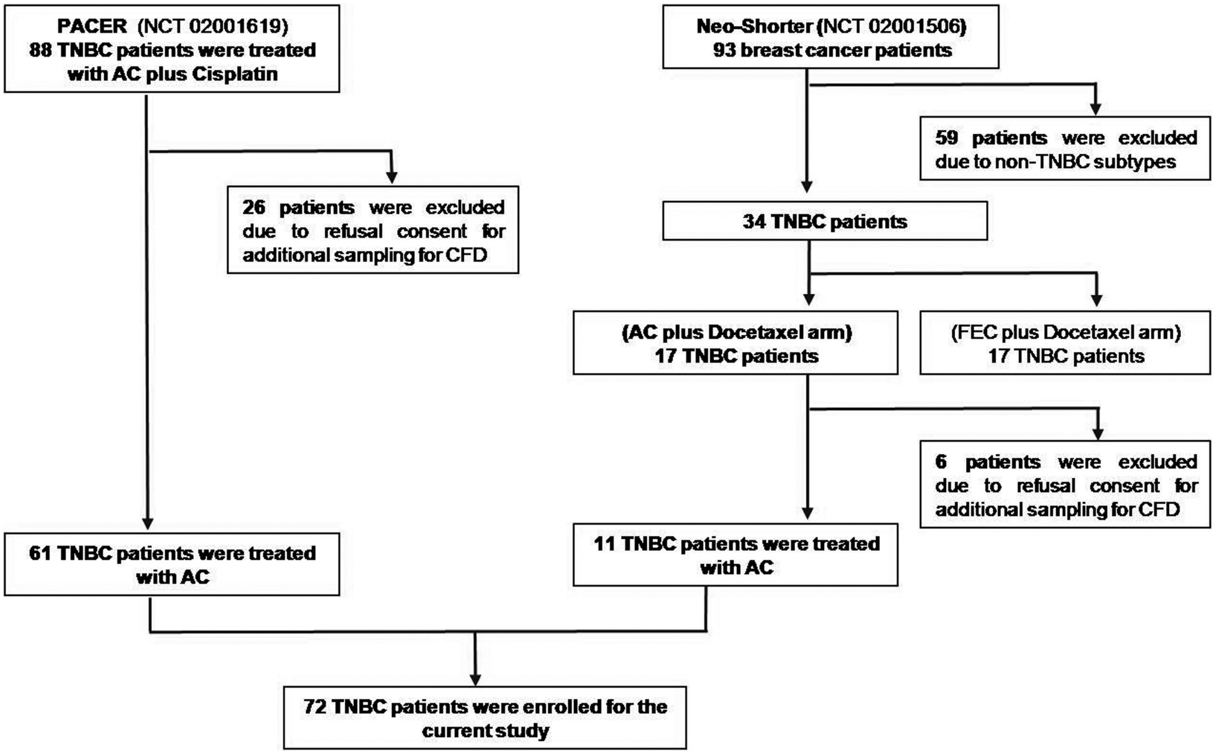

Supplementary Figure 1: Flowchart of patients' enrollment.
